# Catalysis of Rice Straw Hydrolysis by the Combination of Immobilized Cellulase from *Aspergillus niger* on ***β***-Cyclodextrin-Fe_**3**_O_**4**_ Nanoparticles and Ionic Liquid

**DOI:** 10.1155/2015/409103

**Published:** 2015-03-22

**Authors:** Po-Jung Huang, Ken-Lin Chang, Jung-Feng Hsieh, Shui-Tein Chen

**Affiliations:** ^1^Institute of Biological Chemistry, Academia Sinica, 128 Section 2, Academia Road, Nankang, Taipei 115, Taiwan; ^2^School of Environmental Science and Engineering, Guangdong University of Technology, Guangzhou 510006, China; ^3^Department of Food Science, Fu Jen Catholic University, Xinzhuang, Taipei 242, Taiwan; ^4^Institute of Biochemical Sciences, College of Life Science, National Taiwan University, Taipei 115, Taiwan

## Abstract

Cellulase from *Aspergillus niger* was immobilized onto *β*-cyclodextrin-conjugated magnetic particles by silanization and reductive amidation. The immobilized cellulase gained supermagnetism due to the magnetic nanoparticles. Ninety percent of cellulase was immobilized, but the activity of immobilized cellulase decreased by 10%. In this study, ionic liquid (1-butyl-3-methylimidazolium chloride) was introduced into the hydrolytic process because the original reaction was a solid-solid reaction. The activity of immobilized cellulase was improved from 54.87 to 59.11 U g immobilized cellulase^−1^ at an ionic liquid concentration of 200 mM. Using immobilized cellulase and ionic liquid in the hydrolysis of rice straw, the initial reaction rate was increased from 1.629 to 2.739 g h^−1^ L^−1^. One of the advantages of immobilized cellulase is high reusability—it was usable for a total of 16 times in this study. Compared with free cellulase, magnetized cellulase can be recycled by magnetic field and the activity of immobilized cellulase was shown to remain at 85% of free cellulase without denaturation under a high concentration of glucose (15 g L^−1^). Therefore, immobilized cellulase can hydrolyze rice straw continuously compared with free cellulase. The amount of harvested glucose can be up to twentyfold higher than that from the hydrolysis by free cellulase.

## 1. Introduction

Recently, magnetic nanoparticles (MNPs) have been applied to enzyme immobilization because the high surface areas of such particles at the nanometer scale are beneficial to enzyme loading. In contrast, porous carrier materials would limit the rate of diffusion during enzymatic reactions [1], thus reducing the rate of reaction. The most common method for producing synthetic MNPs is the coprecipitation of ferrous and ferric ions at a molar ratio of 1 : 2 by alkali solutions. The reaction is indicated as follows:

(1) $$\begin{array}{c} {\text{Fe}}^{2+}+2{\text{Fe}}^{3+}+8{\text{OH}}^{-}⟶{\text{Fe}}_{3}{\text{O}}_{4(\text{s})}+4{\text{H}}_{2}\text{O} \end{array}$$

Although this method of preparing MNPs is well known, the phase and size of MNPs are difficult to control. The properties of a nanomagnetite can be regulated by (1) temperature [2–4], (2) initial pH [2, 5], (3) stirring velocity [6], (4) different iron salt solutions with varying ratios of ferrous and ferric ions [3, 7], (5) types and concentrations of alkali [3, 8], (6) surfactants [5], and (7) ionic strength of the solution [4].

Immobilization of cellulase on MNPs allows for the recycling of the most costly ingredient of the rice straw hydrolysis process. A variety of methods [9] can be used to immobilize enzymes, including (1) physical adsorption to a solid phase [10], (2) covalent bonding to a solid phase [11], (3) covalent bonding to a soluble polymer [12, 13], (4) cross-linking with bifunctional reagents [14], (5) inclusion in a gel phase [12], and (6) encapsulation [15]. A popular technique for coupling magnetite with cellulase involves the use of silane as a linker. A functional silane compound, such as 3-aminopropyltriethoxysilane (APTES), can self-assemble onto the surface of a magnetite and form covalent bonds [16–18]. Subsequently, the functional silane can transfer the surface of magnetite from dense hydroxyl group to amino group [19]. Therefore, modified nanomagnetite can conjugate with cellulase easily. Garcia III et al.  [20] used *γ*-glycidoxypropyltrimethoxysilane and 3-aminopropyltriethoxysilane that were refluxed in toluene to modify the surface of magnetite. Additionally, they used PEG and PVA as ligands to improve upon the direct attachment on silanized magnetite. As a result, cellulase was immobilized onto the surface of magnetite successfully and the activity of cellulase was comparable to that of free cellulase in a phosphate buffer of pH 5.5. Furthermore, the cellulase could be immobilized onto MNPs using carbodiimide as the coupling agent [21, 22]. Their method of immobilization preserved both cellulase activity and particle size.

The purpose of this study was to immobilize cellulase from* Aspergillus niger* onto aqueous MNPs, which were modified and size-limited by *β*-cyclodextrin. After cellulase was bound to MNPs via 3-aminopropyltriethoxysilane (APTES) and glutaraldehyde directly, we determined the optimum operating conditions. The size and structure of MNPs were measured by Zeta-sizer and X-ray diffraction (XRD). The structure and morphology of immobilized cellulase were confirmed using X-ray photoelectron spectroscopy (XPS) and transmission electron microscopy (TEM).

## 2. Materials and Methods

### 2.1. Chemicals and Raw Materials

Ferrous chloride tetrahydrate, ferric chloride, oleic acid, kerosene, and *β*-cyclodextrin (all from Sigma Chemical Co., USA) and ammonium (J.T.-Baker, USA) were used to synthesize magnetic nanoparticles. Ethanol (J.T.-Baker, USA) was added to stabilize and suspend the magnetic nanoparticles during the silanization of 3-aminopropyltriethoxysilane (APTES, Sigma Chemical Co., USA). Glutaraldehyde (Sigma Chemical Co., USA) was used as the linker between cellulase and the magnetic nanoparticles. The Schiff base was reduced by sodium cyanoborohydride (Sigma Chemical Co., USA).

Acetate buffer (pH 5.5) (J.T.-Baker, USA) was prepared by dissolving acetic acid sodium acetate trihydrate in Milli-Q water. Cellulase from* Aspergillus niger* (Sigma Chemical Co., USA) was used for the hydrolysis of untreated rice straw. The enzymatic activity of cellulase was determined by the method of Ratanakhanokchai et al. [23]. The hydrolysis reaction was carried out by incubation of untreated rice straw with 150 U cellulase per gram of rice straw in 10 mL of 50 mM acetate buffer solution at pH 5.5 and 37°C. A buffer solution of 0.01% sodium azide (Sigma Chemical Co., USA) was added to prevent microorganism contamination. The hydrolytic mixture was incubated in a rotating incubator at 1.67 Hz. After incubation, samples were collected and centrifuged for sugar analysis.

Rice straws from 5-month-old plants of a japonica rice (*O. sativa* L.) variety, Tainung 67, were obtained from the experimental farm (25° 02′ 32.79′′N, 121° 36′ 47.40′′E with 18 m of elevation) located at the Academia Sinica campus, Taipei, Taiwan. The rice plants were transplanted to the field in the first week of March 2009 and the heading (flowering) appeared in the first week of June 2009. The straws (leaves and stems) were collected after the seeds were harvested in late July. The rice plants were approximately 100 cm tall with approximately 13~15 tillers at the time of harvest. The dried rice straws were then ground into powder and stored in sealed plastic bags at room temperature. The size distribution of the rice straws was determined by mesh sieving, and five fractions were obtained: >300 mm (3.39%); 300~150 mm (47.95%); 150~106 mm (15.52%); 106~75 mm (20.57%;) <75 mm (12.63%).

### 2.2. Preparation of Magnetite Nanoparticles

In this study, naked Fe_3_O_4_ magnetic nanoparticles were prepared by the coprecipitation method. FeCl_3_ (2.6 g, 16 mmol) and FeCl_2_·4H_2_O (1.0 g, 5 mmol) were dissolved in 400 mL deionized water. The mixed solution was placed in an inert environment and purged by nitrogen for 30 minutes to prevent oxidation of the magnetite. The solution containing ferric and ferrous ions was then heated to 60°C, during which the color changed from yellow to orange. After the mixture turned orange, an ammonium solution (1.5 M, 100 mL) was added, inducing an immediate color change from orange to black and the formation of a colloid mixture. After the reduction by the alkali solution, the black colloid was heated to 80°C. Oleic acid was added to the solution, and the solution was heated for another hour until the ammonia evaporated. Subsequently, the solution was cooled to room temperature, and 100 mL kerosene was added to the coated magnetite suspension. The suspension was stirred until most of the black color had been transferred into the kerosene. The kerosene layer was collected, and the water layer was discarded. The kerosene layer was dropped onto a *β*-CDs solution with ratios (v/v) to black colloid of 0.02, 0.1, 0.5, and 1. The structure of the magnetic supporter Fe_3_O_4_-*β*-CDs was determined by XRD and ESCA (Electron Spectroscopy for Chemical Analysis). The particle size was measured by Zeta-sizer.

### 2.3. Cellulase Immobilization

A total of 150 mg Fe_3_O_4_-(*β*-CDs) magnetic nanoparticles were coated with 10% (v/v) 3-aminopropyltriethoxysilane (APTES) overnight at 40°C to form amino-functionalized magnetic nanoparticles (AFMNs). Afterwards, 10% (v/v) glutaraldehyde was added as the coupling agent to react with the AFMNs for another 12 hours. The aldehyde-functionalized magnetic nanoparticles (100 mg) were then incubated with cellulase (68 mg) from* Aspergillus niger* in a sodium cyanoborohydride (100 mg) solution to immobilize the cellulase onto the surface of magnetite. The immobilized cellulase was characterized by ESCA (Electron Spectroscopy for Chemical Analysis) and SQUID (Superconducting Quantum Interference Device). The activity of immobilized cellulase was analyzed by the FPU method.

### 2.4. Characterization of Nanoparticles

X-ray diffraction (XRD) patterns were recorded using a PANalytical X' Pert PRO diffractometer with the Cu K*α* line (1.54 Å). Beam divergence was restricted with a 0.15 mm silt on the source side. The X-ray diffraction studies were performed in the scan range of 2*θ* = 15~70° with a scan speed of 1° min^−1^ and a step size of 0.02°. Analysis of the X-ray photoelectron spectra (XPS) was performed on a thermo-ESCLAB 250 using an incident X-ray radiation (Cu K*α*) as the excitation source. Particle size distribution and morphology were analyzed by a vibrating sample magnetometer at room temperature. Fourier transform infrared (FT-IR) spectra were recorded in the wave length rage of 4000~500 cm^−1^ using a Nicolet (Madison, WI) FT-IR spectrometer (model impact 410).

### 2.5. Sugar Determination and Cellulase Activity Measurements

The reducing sugars from hydrolyzed rice straw were measured by the dinitrosalicylic acid method (DNS) [24], and the activities of free and immobilized cellulase were determined by the FPU method. Immobilized cellulase was added to 5 mg filter paper (1 mm width × 6 mm length) overlaid with 150 *μ*L of acetate buffer (50 mM, pH 5.5). The mixture was then incubated at 37°C. After 1 hour, 100 *μ*L of sample was collected from the mixture and 300 *μ*L of DNS reagent was added to this aliquot. The sample was heated at 95°C for 5 min to allow color formation. When the reaction ended, deionized water (600 *μ*L) was added into the solution. The concentration of reducing sugar was determined at 540 nm on a HITACHI UV2010/3010.

## 3. Results and Discussion

### 3.1. Characteristics of Magnetic Nanoparticles

**Figure 1:**
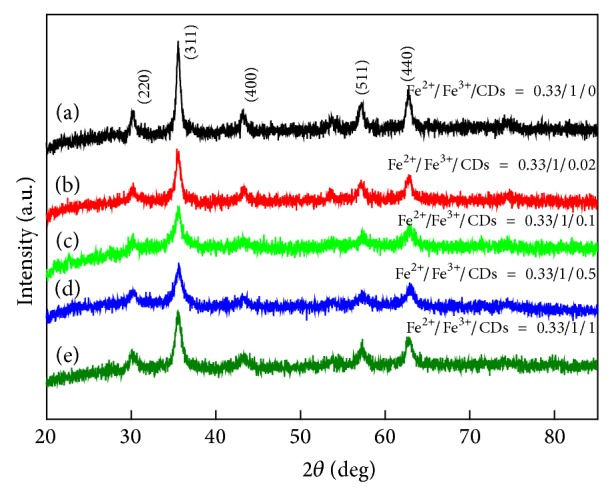
X-ray diffraction patterns of Fe_3_O_4_ with different ratios of ferric ion, ferrous ion, and *β*-CDs: (a) Fe^2+^/Fe^3+^/*β*-CDs = 0.33/1/0, (b) Fe^2+^/Fe^3+^/*β*-CDs = 0.33/1/0.02, (c) Fe^2+^/Fe^3+^/*β*-CDs = 0.33/1/0.1, (d) Fe^2+^/Fe^3+^/*β*-CDs = 0.33/1/0.5, and (e) Fe^2+^/Fe^3+^/*β*-CDs = 0.33/1/1.

**Figure 2:**
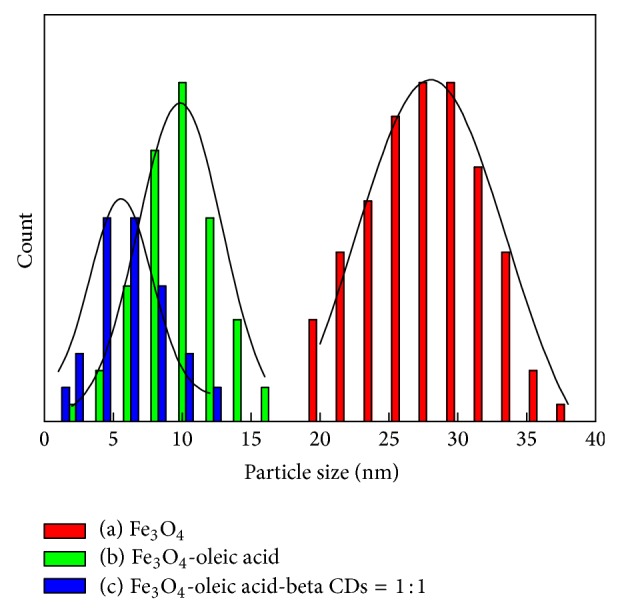
The particle size distributions of Fe_3_O_4_ that were synthesized under different conditions: (a) Fe_3_O_4_; (b) Fe_3_O_4_-oleic acid; (c) Fe_3_O_4_-oleic acid-(*β*-CDs): Fe^2+^/Fe^3+^/*β*-CDs = 0.33/1/1.

XRD was conducted to identify the MNPs that were prepared with ferrous and ferric salts in varying concentrations of *β*-cyclodextrin. Five samples were produced with the following compositions: Fe^2+^/Fe^3+^/CDs = 0.33/1/0, Fe^2+^/Fe^3+^/CDs = 0.33/1/0.02, Fe^2+^/Fe^3+^/CDs = 0.33/1/0.1, Fe^2+^/Fe^3+^/CDs = 0.33/1/0.5, and Fe^2+^/Fe^3+^/CDs = 0.33/1/1 (Figure 1). Other common coprecipitates, such as Fe(OH)_3_ or Fe_2_O_3_, were not observed because the positions of the main peaks only matched well with those from the JCPDS card (19-0629) for Fe_3_O_4_. Our method of MNP preparation yielded highly pure Fe_3_O_4_ particles (Figure 2). Size measurement by the Zeta-sizer revealed that the average size of the Fe_3_O_4_ particles was 28.05 nm. The addition of oleic acid as a surfactant led to a decrease in particle size down to 10.1 nm. The subsequent addition of *β*-cyclodextrin to the samples with Fe^2+^/Fe^3+^/CDs ratios of 0.33/1/0.1 and 0.33/1/1 further reduced the average particle size down to 6.2 and 4.5 nm, respectively. Notably, as the *β*-cyclodextrin concentration increased, the size of the obtained Fe_3_O_4_ crystallite decreased. These results showed that *β*-cyclodextrin can be used to effectively limit the particle size of magnetite.

**Figure 3:**
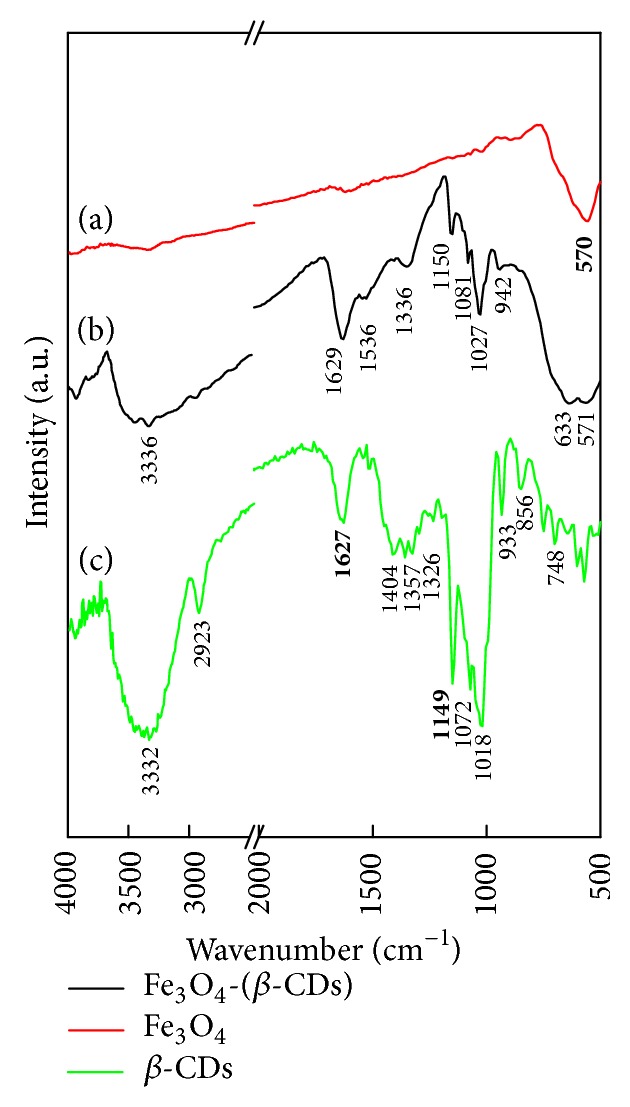
FTIR spectra of (a) Fe_3_O_4_, (b) Fe_3_O_4_-(*β*-CDs): Fe^2+^/Fe^3+^/*β*-CDs = 0.33/1/1, and (c) *β*-CDs.

The Fourier transform infrared (FTIR) spectra of *β*-CDs, Fe_3_O_4_, and *β*-CDs-modified Fe_3_O_4_ revealed the following features (Figure 3). For the Fe_3_O_4_ magnetite, a peak at approximately 570 cm^−1^ was assigned to the stretching vibration of Fe–O (Figure 3(a)). The *β*-CDs spectrum showed large and abroad peaks at approximately 3344 cm^−1^ that corresponded to the O–H stretching vibration (Figure 3(c)). The peaks at approximately 2923 and 2854 cm^−1^ of the *β*-CD spectrum were attributed to the stretching vibration of C–H. Furthermore, the stretching vibrations of the glucose ring, such as OCH, HCH, CCH, and COH, were observed at approximately 1408, 1368, and 1332 cm^−1^. In addition, three peaks at 1156, 1080, and 1028 cm^−1^ were indicative of the C–O–C linkage. For the Fe_3_O_4_-(*β*-CDs) conjugate, two specific peaks of oleic acid were identified with the C=O symmetric stretching vibration at 1708 cm^−1^, the C=C stretching vibration at 1641 cm^−1^, and the =CH bending vibration at 1004 cm^−1^ (Figure 3(b)). Moreover, the conjugation between Fe_3_O_4_ and *β*-CDs was evident from the observation that the serial peaks of *β*-CDs shifted to higher wavenumbers of approximately 6–15 cm^−1^. Therefore, the collective results of ESCA and FTIR indicated that Fe_3_O_4_ conjugated with *β*-CDs covalently.

### 3.2. Characteristics of Immobilized Cellulase on Magnetic Nanoparticles

**Figure 4:**
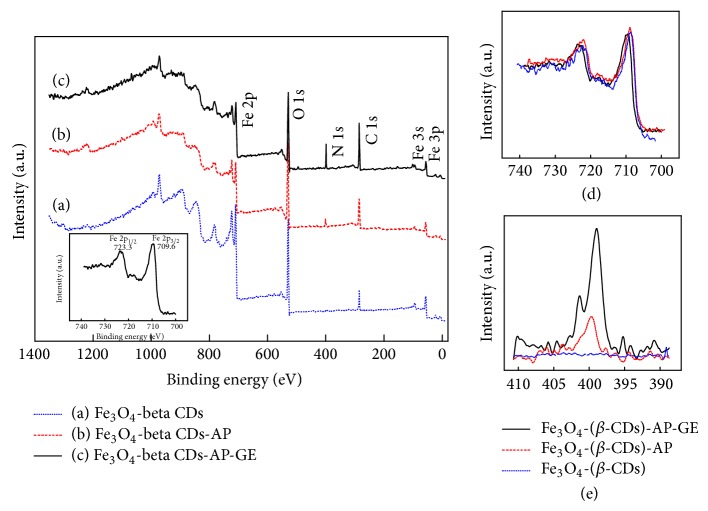
XPS spectra of total binding energies of immobilized cellulase from* Aspergillus niger* on Fe_3_O_4_ nanoparticles: (a) Fe_3_O_4_-(*β*-CDs); (b) Fe_3_O_4_-(*β*-CDs)-AP; (c) Fe_3_O_4_-(*β*-CDs)-AP-GE; (d) binding energy of Fe 2p; (e) binding energy of N 1s; (f) binding energy of C 1s. Dash line: Fe_3_O_4_-(*β*-CDs); dotted line: Fe_3_O_4_-(*β*-CDs)-AP; solid line: Fe_3_O_4_-(*β*-CDs)-AP-GE. The Fe_3_O_4_ MNPs were produced using the following ratio of ions and *β*-CDs: Fe^2+^/Fe^3+^/*β*-CDs = 0.33/1/1.

The measurement of total binding energy of Fe_3_O_4_-(*β*-CDs) showed characteristic peaks of Fe at 708.2 and 722 eV, which were the binding energy of Fe 2p 3/2 and Fe 2p 1/2, respectively (Figure 4(a)). When cellulase was immobilized onto Fe_3_O_4_-(*β*-CDs) after APTES coating, the binding energy of Fe was shifted higher by 1-2 eV (Figure 4(d)). This upward shift was due to the transfer of the Fe electron density to cellulase, thus making it more difficult to emit the Fe electrons. Moreover, compared with Fe_3_O_4_-(*β*-CDs)-AP and Fe_3_O_4_-(*β*-CDs)-APGE (Figures 4(b) and 4(c)), the characteristic peak of N was clearly detected and its intensity increased after immobilization. When APTES was conjugated onto the surface of magnetite, we detected the amine functional group at approximately 400.4 eV (Figure 4(e)). After immobilization of cellulase, two peaks were shown in the spectrum of Fe_3_O_4_-(*β*-CDs)-APGE, with one at 399.8 eV, which indicated the formation of Schiff base, and the other at 401.2 eV, which corresponded to the free amine of cellulase.

**Figure 5:**
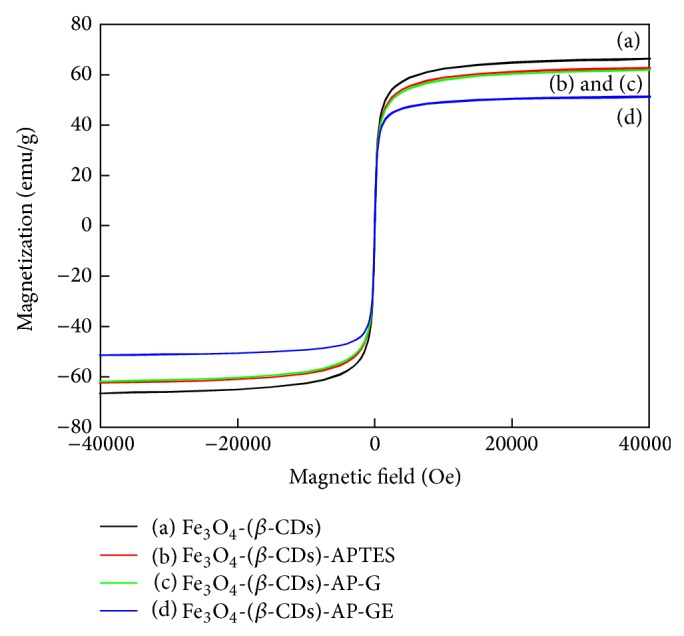
Magnetization curves and coercivity measurements at 300 K of Fe_3_O_4_ nanoparticles at different stages of synthesis and modification up to the immobilization of cellulase: curve (a) represents *β*-CD-modified Fe_3_O_4_; curve (b) represents APTES-modified particles; curve (c) represents glutaraldehyde-conjugated particles; curve (d) represents cellulase-bound particles.

**Figure 6:**
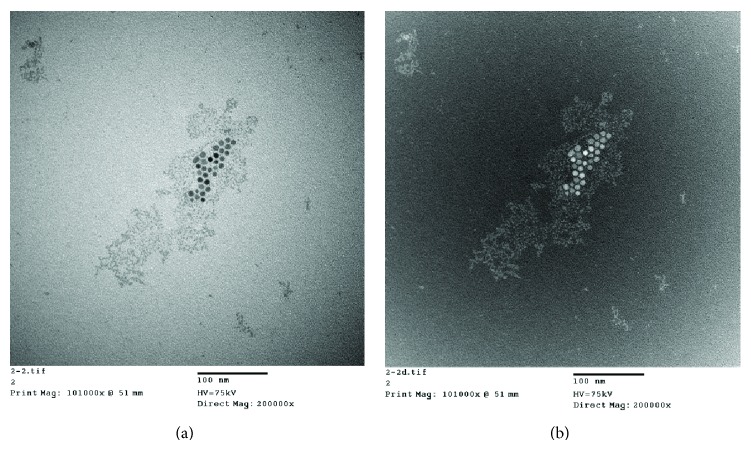
Morphology of immobilized cellulase. (a) TEM bright field (×200 k) and (b) TEM dark field (×200 k).

**Figure 7:**
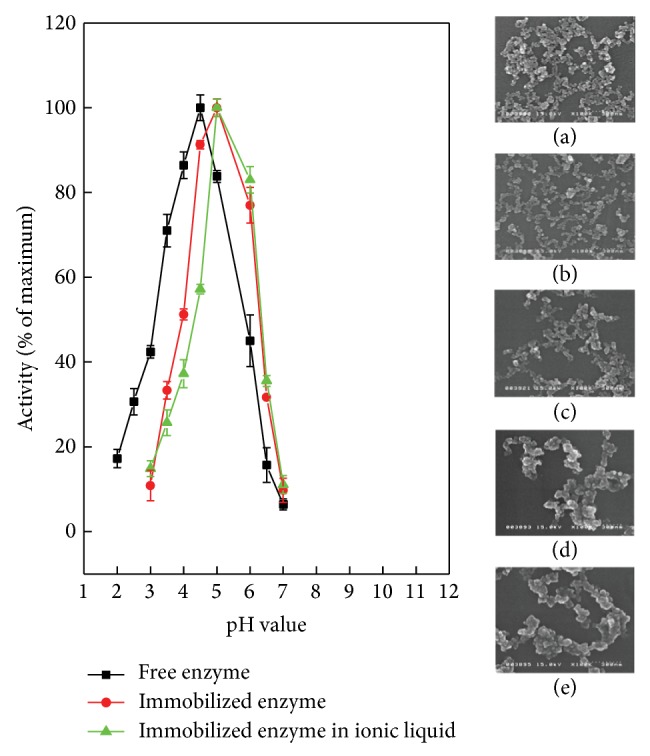
Effect of pH on the activity of (■) free cellulase, (●) immobilized cellulase, and (▲) immobilized cellulase + ionic liquid. Morphologies of immobilized cellulase under different pH were analyzed by SEM: (a) original immobilized cellulase; (b) pH = 5.0; (c) pH = 4.0; (d) pH = 3.0; (e) pH = 2.0.

The magnetic properties of the magnetite nanoparticles were measured by the Quantum Design MPMS-XL7 magnetometer with the application of field dependence of magnetization. The values of saturation magnetization and coercivity are shown in Figure 5 and tabulated in Table 1. The nanoparticles at each step were superparamagnetic in nature. However, the saturation magnetization value was gradually reduced from 64.7 to 50.7 emg g^−1^ and the coercivity value was gradually increased from 0.288 to 3.82 Oe when the nanomagnetite was modified stepwise from Fe_3_O_4_-(*β*-CDs) to Fe_3_O_4_-(*β*-CDs)-AP-GE, respectively. In other words, when cellulase was immobilized onto Fe_3_O_4_-(*β*-CDs), the barrier of magnetite was produced by each following modification. Morphological analysis of immobilized cellulase by TEM (Figure 6) and SEM (Figure 7) showed that the average particle size was approximately 30 nm.

### 3.3. Characteristics of Immobilized Cellulase on Magnetic Nanoparticles

Maximum and optimum amount of grafting (i.e., the production of Fe_3_O_4_-(*β*-CDs)-AP-GE) was achieved with the combination of 10% (v/v) APTES, 10% (v/v) glutaraldehyde, and 2.4% (wt) cellulase, when the dose of Fe_3_O_4_-(*β*-CDs) was 10 mg/mL. The activity of cellulase was reduced from 60.6 to 58.9 U g^−1^ enzyme after immobilization in acetate buffer (pH 5.5), which was 97% of the activity of free cellulase. The optimum operating condition was changed from acidic to weakly acidic condition. Previous studies on the immobilization of cellulase from* Trichoderma viride* onto Fe_3_O_4_ also showed that the optimum condition of cellulase activity shifted from acidic to weakly acidic condition [20, 21]. This change is beneficial for immobilized cellulase using magnetite as the supporter because magnetite is more prone to erosion under acidic conditions, even though an acidic condition is more suitable for free cellulase. The phase transformation was previously demonstrated [25–27], and the structure was shown to be destroyed under acidic conditions. This result was presented by SEM in Figure 7. The original immobilized cellulase had a particle size of approximately 20~30 nm and the boundary was clearly identifiable (Figure 7(d)). At pH 5.0, the surface structure of the magnetic particles was still maintained. However, at lower pH values the particle surface was eroded by the acetic acid. As the pH was lowered, we observed a surface phase transition from a smooth state to a more uneven state, and the properties of magnetization and supermagnetism were reduced or completely eliminated by acid erosion. Therefore, the weakly acidic condition is more suitable for hydrolysis catalyzed by immobilized cellulase on a magnetic supporter.

In the hydrolysis experiment, the initial rate of rice straw hydrolysis was reduced from 2.26 to 1.553 g L^−1^ hr^−1^ when cellulase was added at a concentration of 150 U g^−1^ rice straw. The ionic liquids could be used to dissolve cellulose by disrupting hydrogen bonding within cellulose [28–30]. SEM results from previous studies showed that ionic liquids not only removed the outer structural boundaries of cellulose but also improved its hydrophilicity [29, 30]. Zhao et al. [30] reported that these actions of ionic liquids could enhance cellulose hydrolysis. The hydrolysis rate would increase by fourfold compared with no ionic liquid treatment. Therefore, we tested the effects of three ionic liquids including 1-butyl-3-methylimidazolium chloride ([bmim]Cl), 1-butyl-3-methylimidazolium hydrogen sulfate ([bmim]HSO_4_), and 1-ethyl-3-methylimidazolium diethyl phosphate ([emim]PO_4_(C_2_H_6_)_2_) on cellulose hydrolysis.

**Figure 8:**
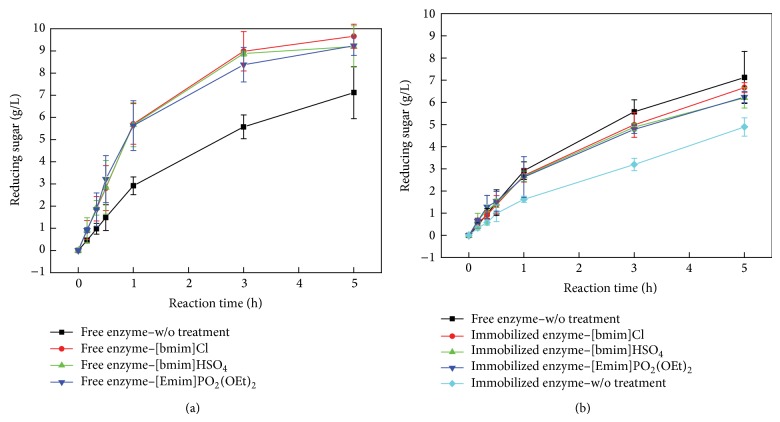
The concentration of reducing sugar that was produced from rice straw (150 U g^−1^) hydrolysis in the presence of different ionic liquids at pH 5.0, 37°C, and 1.67 Hz rotation speed. (a) Free cellulase with or without ionic liquid; (b) immobilized cellulase with or without ionic liquid. ■: rice straw hydrolysis by cellulase without ionic liquid treatment; ●: rice straw hydrolysis by cellulase and 1-butyl-3-methylimidazolium chloride ([bmim]Cl); ▲: rice straw hydrolysis by cellulase and 1-butyl-3-methylimidazolium hydrogen sulfate ([bmim]HSO_4_); ▼: rice straw hydrolysis by cellulase and 1-ethyl-3-methylimidazolium diethyl phosphate ([emim]PO_4_(C_2_H_6_)_2_); ◆: rice straw hydrolysis by immobilized cellulase without ionic liquid treatment.

The values of the initial rate of hydrolysis and the amount of reducing sugar are shown in Figure 8 and tabulated in Table 2. All three ionic liquids increased the amount of reducing sugar (11.9–14.16 g L^−1^) compared with the control condition with free cellulase only (5.97 g L^−1^). Additionally, the initial rate of hydrolysis was increased from approximately 2.918 to 5.7 g L^−1^ h^−1^ after adding any of the ionic liquids. Even though immobilized cellulase was not as active as free cellulase in rice straw hydrolysis, this difference was compensated by adding ionic liquid to the reaction with the immobilized enzyme. Using immobilized cellulase, the amount of sugar produced was increased from 3.58 to 5.3 g L^−1^ h^−1^ and the initial rate of hydrolysis was increased from 1.6 g L^−1^ h^−1^ to 2.7 g L^−1^ h^−1^ on average when an ionic liquid was introduced.

**Figure 9:**
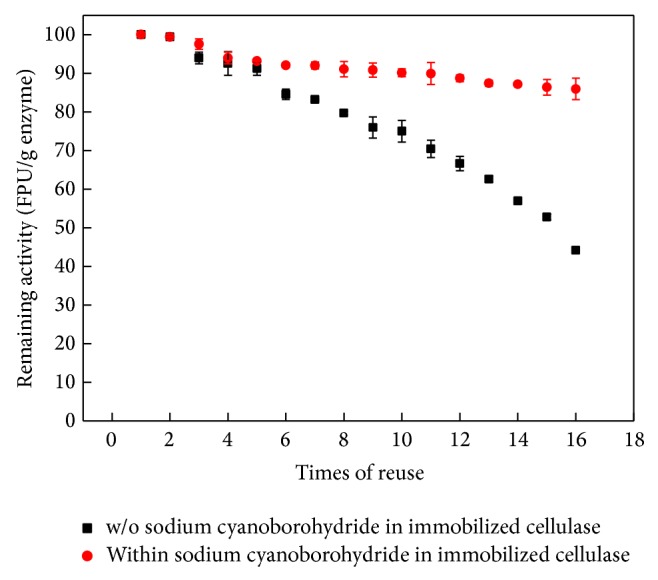
Activities of immobilized cellulase after repeated use.

When cellulase was immobilized onto Fe_3_O_4_, it could be collected by magnet and reused. We found that immobilized cellulase was still active after 16 rounds of hydrolysis, with each round set for 12 hours (Figure 9). The activity of immobilized cellulase remained at 44.15% of the initial activity after 16 cycles. Furthermore, the addition of sodium cyanoborohydride during the process of immobilization could trigger reductive amidation to convert Schiff's base to a carbon-nitrogen single bond, which is more stable than Schiff's base. We assessed the activities of recycled cellulase with or without the inclusion of sodium cyanoborohydride and found that, after 16 rounds of hydrolysis, the sodium cyanohydride-containing samples retained 85% of the initial cellulase activity, while the control group without sodium cyanoborohydride only retained approximately 40% of the initial activity. Thus, the total amount of sugar from rice straw hydrolysis was ten- to twentyfold higher when immobilized cellulase was used instead of free cellulase.

## 4. Conclusions

In this study, we found that *β*-CDs could effectively decrease the size of MNPs. The particles were reduced from 28.05 to 4.5 nm in diameter and became more uniformly distributed compared with those that were not conjugated to *β*-CDs. Enzymatic attachment was confirmed by XPS. After immobilization, the characteristic peak of iron was shifted to a higher binding energy by 1-2 eV. Moreover, the characteristic peak of N was detected at 399.2 eV (Schiff's base) and 401.4 eV (free amine). These data suggested that cellulase was successfully immobilized onto the surface of nanomagnetite using the silane system. The immobilized cellulase not only retained the activity of free cellulase but also gained the properties of magnetization, specifically supermagnetism. The optimum pH for the enzyme shifted from 4.0 to 5.5 after immobilization. This higher pH serves to stabilize the magnetite-associated cellulase during hydrolysis because the decrease in acidity prevents structural damage to the magnetite.

Although the activity of immobilized cellulase was reduced by 10% compared with free cellulase, the addition of an ionic liquid, such as 1-butyl-3-methylimidazolium chloride ([bmim]Cl), 1-butyl-3-methylimidazolium hydrogen sulfate ([bmim]HSO_4_), and 1-ethyl-3-methylimidazolium diethyl phosphate ([emim]PO_4_(C_2_H_6_)_2_), could enhance rice straw hydrolysis by immobilized cellulase. The initial rate and amount of sugar production were both increased by adding ionic liquids, and the activity of immobilized cellulase was enhanced to match that of free cellulase.

The immobilized cellulase was shown to be reusable for 16 cycles of hydrolysis while still maintaining approximately 80% residual activity. Until now, the total concentration of sugar was 208.92 g L^−1^ (Table 3), which is higher than using the free enzyme (10.5 g L^−1^). Collectively, findings from this study suggest that immobilization of cellulase may greatly benefit the ethanol production industry.

## Figures and Tables

**Table 1:** Saturation magnetization and coercivity values at 300 K of Fe_3_O_4_ nanoparticles synthesized with various iron salts.

Type of magnetic nanoparticles	Saturation magnetization (emg g^−1^)	Coercivity (Oe)
Fe_3_O_4_-(*β*-CDs)	64.7	0.288
Fe_3_O_4_-(*β*-CDs)-APTES	61.3	0.330
Fe_3_O_4_-(*β*-CDs)-AP-G	60.5	0.479
Fe_3_O_4_-(*β*-CDs)-AP-GE	50.7	3.82

**Table 2:** The amount of sugar produced and the initial rate of reducing sugar formation by free and immobilized cellulase with or without ionic liquid treatment.

Type of enzyme	Amount of sugar (g L^−1^) (24 hours)	Initial rate (g L^−1^ h^−1^)
Free cellulase (FC)	5.97 ± 1.29	2.918 ± 1.89
FC + [bmim]Cl	11.91 ± 3.33	5.807 ± 3.66
FC + [bmim]HSO_4_	13.56 ± 4.27	5.671 ± 1.06
FC + [emim]PO_4_(C_2_H_6_)_2_	14.16 ± 1.82	5.635 ± 2.57
Immobilized cellulase onto Fe_3_O_4_ (IC)	4.78 ± 2.44	1.629 ± 0.92
IC + [bmim]Cl	5.392 ± 2.07	2.739 ± 1.31
IC + [bmim]HSO_4_	5.821 ± 1.33	2.711 ± 1.55
IC + [emim]PO_4_(C_2_H_6_)_2_	5.719 ± 3.49	2.595 ± 0.84

**Table 3:** The total amount of sugar from rice straw hydrolysis by free cellulase and immobilized cellulase with or without the use of NaBH_3_CN.

Type of enzyme	Total concentration of sugar (g/L)
Free cellulase	10.5 ± 3.58
Immobilized cellulase onto Fe_3_O_4_ (w/o adding NaBH_3_CN)	110.66 ± 21.11
Immobilized cellulase onto Fe_3_O_4_ (add NaBH_3_CN)	208.92 ± 29.77
